# Associations of Breast Milk Microbiota, Immune Factors, and Fatty Acids in the Rat Mother–Offspring Pair

**DOI:** 10.3390/nu12020319

**Published:** 2020-01-25

**Authors:** Ignasi Azagra-Boronat, Alba Tres, Malén Massot-Cladera, Àngels Franch, Margarida Castell, Francesc Guardiola, Francisco J. Pérez-Cano, Maria J. Rodríguez-Lagunas

**Affiliations:** 1 Physiology Section, Department of Biochemistry and Physiology, Faculty of Pharmacy and Food Science, University of Barcelona (UB), 08028 Barcelona, Spain; ignasiazagra@ub.edu (I.A.-B.); malen.massot@ub.edu (M.M.-C.); angelsfranch@ub.edu (À.F.); margaridacastell@ub.edu (M.C.); mjrodriguez@ub.edu (M.J.R.-L.); 2 Nutrition and Food Safety Research Institute (INSA-UB), 08921 Santa Coloma de Gramenet, Spain; atres@ub.edu (A.T.); fguardiola@ub.edu (F.G.); 3 Department of Nutrition, Food Science and Gastronomy, Torribera Food Science Campus, Faculty of Pharmacy and Food Science, University of Barcelona (UB), 08921 Santa Coloma de Gramenet, Spain

**Keywords:** breast milk, rat, fatty acids, immune system, microbiota

## Abstract

The present study aimed to analyze the rat breast milk profile of fatty acids (FA), immunoglobulins (Ig), microbiota, and their relationship, and to further assess their associations in the mother–offspring pair. Dams were monitored during the three weeks of gestation, allowed to deliver at term, and followed during two weeks of lactation. At the end of the study, milk was obtained from the dams for the analysis of fatty acids, microbiota composition, immunoglobulins, and cytokines. Moreover, the cecal content and plasma were obtained from both the dams and pups to study the cecal microbiota composition and the plasmatic levels of fatty acids, immunoglobulins, and cytokines. Rat breast milk lipid composition was ~65% saturated FA, ~15% monounsaturated FA, and ~20% polyunsaturated FA. Moreover, the proportions of IgM, IgG, and IgA were ~2%, ~88%, and ~10%, respectively. Breast milk was dominated by members of *Proteobacteria*, *Firmicutes,* and *Bacteroidetes* phyla. In addition, forty genera were shared between the milk and cecal content of dams and pups. The correlations performed between variables showed, for example, that all IgGs subtypes correlated between the three compartments, evidencing their association in the mother-milk-pup line. We established the profile of FA, Ig, and the microbiota composition of rat breast milk. Several correlations in these variables evidenced their association through the mother-milk-pup line. Therefore, it would be interesting to perform dietary interventions during pregnancy and/or lactation that influence the quality of breast milk and have an impact on the offspring.

## 1. Introduction

Maternal milk is a complex fluid that supports the growth and development of infants in early life [1]. Beyond the nutritional composition, maternal milk is a source of numerous bioactive substances, such as immune factors, hormones, microbial derivatives, or enzymes, which are needed for adequate intestinal function and immune homeostasis [2].

Lipids comprise the second largest fraction of breast milk, providing the newborn with energy, essential polyunsaturated fatty acids (PUFA), and fat-soluble vitamins [3]. Milk lipids are primarily found as triacylglycerols inside fat globules, which are formed in the mammary gland from fatty acids (FA) taken up from the circulation or synthesized in situ from glucose [4,5]. The maternal diet influences substantially the composition of FA in human milk, with special interest on the content of the mono-unsaturated fatty acids (MUFA) oleic acid (OA, 18:1 *n*-9) and the essential PUFA linoleic acid (LA, 18:2 *n*-6) and α-linolenic acid (ALA, 18:3 *n*-3) [5,6]. Variations in FA content are also found over the course of the day (i.e., daytime or nighttime) and over the time of each breastfeeding meal (i.e., foremilk and hindmilk) [3,4]. 

Human milk also contains long-chain PUFA (LC-PUFA), such as arachidonic acid (AA, 20:4 *n*-6), eicosapentaenoic acid (EPA, 20:5 *n*-3), or docosahexaenoic acid (DHA, 22:6 *n*-3). These FA have an important role to achieve full development of brain, retina, and other organs. However, although they are not essential in adults, due to their slow synthesis and the high requirements in early life, their presence in breast milk is of importance for the nutrition of the newborn [7,8]. LC-PUFA are also known to be modulators of inflammation and immunity, influencing lymphocyte proliferation, the production of cytokines, and natural killer cell activity, among others [9,10,11]. Accordingly, AA is a well-known precursor of eicosanoids, promoting inflammatory processes, whereas LC *n*-3 PUFA, such as EPA, are known to inhibit the immune response [10]. Thus, an imbalanced *n*-6/*n*-3 ratio toward *n*-6 is highly inflammatory and may contribute to a range of chronic diseases, such as obesity, atherosclerosis, and diabetes [12,13].

Neonates are born with an immature immune system, unable to cope with all the threads they encounter in the external environment. In order to compensate this, the offspring acquire passive immunity via the immune factors transmitted through transplacental transfer during gestation and through breast milk after birth [14]. In this context, maternal milk has high levels of immunoglobulins (Ig), with dominance of secretory IgA in humans, which arises from entero-mammary cell circulation and protects the infant against enteric infections [15]. Moreover, other immune factors provided in breast milk such as cytokines, growth factors, immune cells, lactoferrin, oligosaccharides, or antioxidants also participate actively in the development of an independent immune system of the newborn [9].

The colonization of the gut occurs when the infant is born and enters the highly contaminated extrauterine environment [16]. Breast milk is a major determinant of adequate colonization, not only because of the selective pressure that it exerts as a diet but also because it has been proved to have its own microbiota [17]. In this regard, it has been proposed in humans that certain bacteria from the intestine of the mother could translocate to the mammary glands reaching the breast milk, constituting the human milk microbiota, and, thereafter, they could be transmitted to the newborn [18]. Moreover, the indigestible oligosaccharides present in breast milk have a direct effect on shaping the microbiota of the infant [19]. Therefore, achieving a balanced intestinal microbial ecosystem is important for the development and maintenance of correct intestinal and immune functions [14]; a disturbance of its balance, known as dysbiosis, could lead to immune and metabolic diseases at short-term or later in life [17].

On the whole, breast milk has been identified as having multiple health-promoting effects in the newborn, in terms of supporting normal development and intestinal function. Key elements contributing to these are FA, immune factors, and microbiota, which are strongly influenced by the diet of the mother. Rodents are widely used as laboratory animals to study pre- and postnatal physiological development or as models for dietary interventions in early life, either through supplementation of the dams or pups [20]. However, due to the scarce literature available, there is a large unmet need for the establishment of the breast milk profile in rodents, which could help at translational level to understand some human processes in this context. Therefore, the present article aimed to analyze the rat breast milk profile in terms of FA, Ig, microbiota, and their relationship, and to further assess the role they play in the mother–offspring transmission line.

## 2. Materials and Methods

### 2.1. Animals

Wistar adult rats (9 females and 3 males, RjHan:WI) were obtained from Janvier Labs (Le Genest-saint-Isle, France) and individually housed in cages containing large fibrous particles bedding. Dams were given a standard diet corresponding to the American Institute of Nutrition 93G formulation [21] (Teklad Global Diet 2014, Envigo, Indianapolis, IN, USA) and water ad libitum. An acclimatization period of 7 days allowed the animals to stabilize in the new environment. Then, animals were crossed by placing 2 randomly selected females inside the cage of each of the males during 24 h. Afterward, the females were once again individually housed. The experiment was finally conducted with six pregnant rats, which were monitored daily and allowed to deliver naturally. The day of birth was established as day 1 of life. On day 2, litters were reduced to 8 pups per lactating dam, with free access to maternal milk and rat diet. Animals were housed under controlled conditions of temperature and humidity in a 12 h light–12 h dark cycle, in the Faculty of Pharmacy and Food Science animal facility (University of Barcelona, Spain). 

All experimental procedures were conducted in accordance with the institutional guidelines for the care and use of laboratory animals and were approved by the Ethical Committee for Animal Experimentation of the University of Barcelona and the Catalan Government (CEEA-UB Ref. 486/16 and DAAM9268, respectively), which are in full compliance with national legislation following the EU-Directive 2010/63/EU for the protection of animals used for scientific purposes.

### 2.2. Experimental Design and Sample Collection

The animals were monitored for 5 weeks, comprising the three weeks of gestation and the first two weeks of lactation. At the end of this period, the dams were intramuscularly anesthetized with 10 mg/100 g ketamine (Merial Laboratories S.A., Lyon, France) and after 30 min 2 UI of oxytocin (Syntocinón 10 U.I./mL, Alfasigma, Bologna, Italia) were intraperitoneally injected. Milking was initiated after 5 min of oxytocin administration, by gentle manual stimulation from the base to the top of the teat. The milk was collected into a pipette tip, which was attached into a manual vacuum device (Nahrinel, Novartis, Basel, Switzerland) [22]. Afterward, dams (*n* = 6) and pups (*n* = 48) were euthanized in order to obtain the blood and the cecal content (CC). The blood was collected in EDTA tubes and then centrifuged in order to obtain plasma. The milk was centrifuged (800× *g*, 10 min) in order to obtain lactic serum. All samples were stored at −80 °C. Plasma and lactic serum samples were used for the quantification of Ig and FA, whereas the CC was used for the analysis of the microbiota composition.

### 2.3. Lipid Extraction, Methylation, and Quantification of FA

The analysis of FA in milk (*n* = 6) and plasma (*n* = 6 in dams and *n* = 24 in pups, 4 pups/litter) was performed as previously described [23], with some adaptations. Briefly, the lipid fraction of the samples was extracted with a mix of CHCl3/MeOH (2:1, v/v; Scharlab S.L., Barcelona, Spain) using a high-speed homogenizer (polytron PT 10-35, Kinematica, Lucerne, Switzerland), and derivatized in order to obtain the fatty acids methyl esters (FAME), which were determined by gas chromatography in an Agilent 4890D chromatograph (Agilent Technologies, Waldbronn, Germany) equipped with a flame ionization detector and a split-splitless injector, set at 300 and 270 °C, respectively. The split ratio was 1:30. The chromatographic separation was performed on an SP-2380 capillary column (60 m, 0.25 mm i.d., 0.2 µm, Supelco, Bellefonte, PA, USA). The oven temperature program was as follows: initial temperature of 150 °C (held at this temperature for 1 min) up to 180 °C at 3 °C/min, from 180 °C (0.5 min) up to 220 °C at 14.5 °C/min, and from 220 °C (3 min) up to 250 °C at 9.9 °C/min, maintaining 9.5 min at 250 °C. The carrier gas was hydrogen (25 p.s.i). For the injection, 2 µL of the samples were used, and the FA were identified by comparing the retention time with a standard mix (Supelco 37 component FAME Mix, Sigma-Aldrich Co., St. Louis, MO, USA). A total of 28 FA were identified. The quantification was performed by peak area normalization (the quantitative results are obtained by expressing the area of a given peak as a percentage of the sum of the areas of all the identified peaks).

### 2.4. Quantification of Ig and Cytokines

The quantification of Ig (IgM, IgG1, IgG2a, IgG2b, IgG2c, and IgA) in plasma of pups (*n* = 16, 2–3 pups/litter) and in plasma and lactic serum of dams (*n* = 6) was performed at the end of the study, as previously described [24]. Briefly, specific color-coded capture beads were bound to the analyte of interest. Then, different detection antibodies conjugated to phycoerythrin (PE) were added. The specific concentration of each analyte was obtained by MAGPIX^®^ analyzer (Luminex Corporation, Austin, TX, USA) at the Cytometry Service of the Scientific and Technological Centres of the University of Barcelona (CCiT-UB). Assay sensitivity was as follows: 0.02 ng/mL for IgM; 0.78 ng/mL for IgG1; 0.02 ng/mL for IgG2a; 0.11 ng/mL for IgG2b; 0.19 pg/mL for IgG2c; and 0.48 pg/mL for IgA. Similarly, the quantification of cytokines (IFNγ, TNFα, IL-4, IL-6, IL-10, and IL-12) in breast milk was performed, as previously described [24].

### 2.5. Microbial DNA Extraction and Sequencing

Genomic DNA was extracted from ~0.5 mL milk samples (*n* = 6) using the DNeasy Blood and Tissue Mini kit (Qiagen, Madrid, Spain) and from randomly selected CC (*n* = 3) of three mother–pup pairs. For that, QIAamp DNA Stool Mini kit (Qiagen) was used, including both enzymatic treatment and mechanical lysis. In order to increase quality and quantity of DNA, the QIAmp Micro kit (Qiagen) was used. Final concentrations after extraction ranged 8.6–16.4 ng/µL for pups’ CC, 208.6–431.8 ng/µL for dams’ CC, and 10.8–30.5 ng/µL for the breast milk. Fifty nanograms of DNA were amplified following the 16S Metagenomic Sequencing Library Illumina 15044223 B protocol (Illumina Inc., San Diego, CA, USA). In brief, in the first amplification step, primers were designed containing a universal linker sequence allowing amplicons for incorporation indexes, sequencing primers by Nextera XT Index kit (Illumina Inc.) and 16S rRNA gene universal primers [25]. In the second and last amplification, indexes were included. Libraries were quantified by fluorimetry using Quant-iT™ PicoGreen™ dsDNA Assay Kit (Thermo Fisher Scientific, Barcelona, Spain) and pooled prior to sequencing on the MiSeq platform (Illumina Inc.), configured at 300 cycles paired reads. The size and quantity of the pool were assessed in the Bioanalyzer 2100 (Agilent Technologies) and with the Library Quantification Kit for Illumina (Kapa Biosystems Inc., Wilmington, MA, USA), respectively. PhiX Control library v3 (Illumina Inc.) was combined with the amplicon library (expected at 20%). Image analysis, base calling, and data quality assessment were performed in the MiSeq instrument.

### 2.6. Processing of Sequences

The software Paired-End read merger (PEAR v 0.9.6, Exelixis Lab, Heidelberg, Germany) was used to merge raw sequences forward and reverse, in order to obtain the complete sequence. Using this approach, the ends of the obtained sequences were overlapped in order to get complete sequences. The amplification primers from the sequences obtained in the sequencing step were trimmed with Cutadapt v1.8.1 [26], using parameters by default, in order to reduce the bias in the annotation step. Once the primers had been removed, sequences lower than 200 nucleotides were excluded from the analysis because short sequences have a higher chance to generate wrong taxonomical groups association. After obtaining the clean complete sequences, a quality filter was applied in order to delete sequences with poor quality. Those bases in extreme positions that did not have Q20 (99%) of well-incorporated bases in the sequencing step or more Phred quality score were removed, and later, sequences which quality mean did not surpass the Q20 threshold, as a mean quality of the whole sequence, were also deleted. The resulting sequences were inspected for PCR chimera constructs that may occur during the different experimental processes, which were removed from further analysis. Each group of sequences was compared to a database of rRNA using an alignment BLAST strategy to associate taxonomic groups. The relative proportions of phyla, families, genera, and species were calculated. 

### 2.7. Analysis of the Microbiota Composition

The qualitative presence or absence of genera was represented in a Venn diagram. One of the six milk samples was contaminated (it contained a high proportion of *Enterobacteriaceae*) and was discarded from the study. All bacterial groups detected in 4 or 5 (out of the 5 samples included) with proportions higher than 0.001% were computed as present, while the bacterial groups detected in 3 or less animals were computed as absent. Similarly, in cecal samples, the presence was established at a cutoff of 2 or higher (out of the 3 samples analyzed).

A Principal Components Analysis (PCA) of the different genera was also performed. To develop the model, Simca v14.1 was used (Umetrics, Umeå, Sweden). Two data matrices were constructed consisting of 11 rows and 166 variables corresponding to the taxonomic analysis of genera. PCA was conducted on both data matrices in order to explore the presence of any natural clustering in the data. In the pre-processing of the PCAs, the mean-centering and unit variance scaling were applied.

### 2.8. Statistical Analysis

The Statistical Package for the Social Sciences (SPSS v22.0) (IBM, Chicago, IL, USA) was used for the statistical analysis. Shapiro–Wilk and Levene tests were used to determine normality and homogeneity of variance of the data, respectively. Student’s *t*-test was used to analyze the differences of FA, Ig, and microbiota among tissues. The Spearman correlation coefficient was used to search for correlation between the variables analyzed. For the correlation analysis, each dam–pup pair was considered separately. Significant differences were established when *p* < 0.05.

## 3. Results

### 3.1. FA Composition

The FA composition was analyzed in the milk and in the plasma of dams and pups (Table 1). The average breast milk saturated fatty acids (SFA) content was ~60% with palmitic acid (PA, 16:0) and myristic acid (MA, 14:0) comprising more than 40%. However, the content of SFA in the plasma of the dams and pups was considerably lower (*p* < 0.05), ~40% of total FA, respectively, being palmitic acid and stearic acid (SA, 18:0) the most abundant.

**Figure 1:**
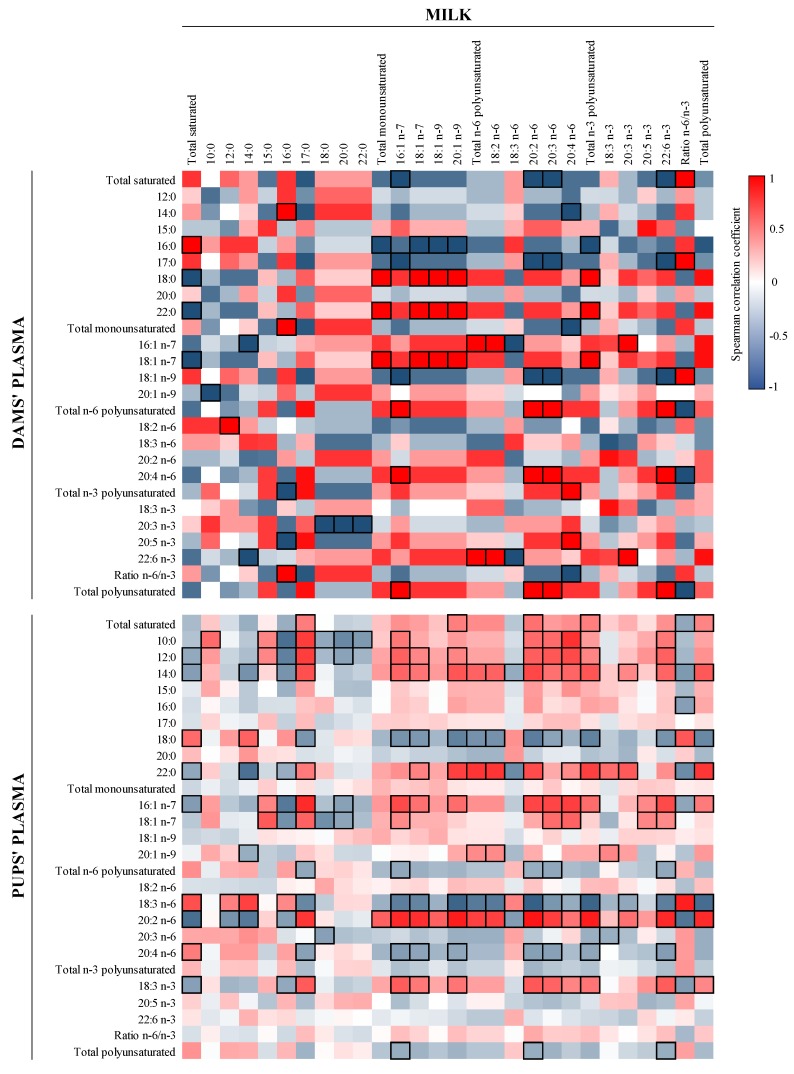
Correlation between the FA composition of milk and that of dams’ and pups’ plasma. The Spearman correlation coefficient is represented in the heat map following the color in the legend. Correlations with statistical significance (*p* < 0.05) are shown in a bold frame. Results derived from *n* = 6 for milk and dams’ plasma and *n* = 24 for pups’ plasma.

The correlations between FA content of milk and plasma of the dams and pups were also studied (Figure 1). Although no significant correlations were detected in matching FA within type of sample (e.g., 16:0 in milk and 16:0 in plasma), many other positive and negative associations appeared. For instance, the MFA in milk correlated positively with dams’ plasma SA, behenic acid (BA, 22:0), and cis-vaccenic acid (CVA, 18:1 n-7) and negatively with PA (*p* < 0.05). In addition, the dams’ plasma total PUFA correlated positively with milk palmitoleic acid (POA, 16:1 n-7), eicosadienoic acid (EDA, 20:2 *n*-6), dihomo-gamma-linolenic acid (DGLA, 20:3 *n*-6), and DHA and, therefore, negatively with the ratio *n*-6/*n*-3 (*p* < 0.05).

When comparing the FA milk profile with that in the pups’ plasma (Figure 1), such correlations were not as strong as those between milk and the dams’ plasma. However, some significant correlations were found in matching FA, which were positive for capric acid (CA, 10:0), POA, γ-linoleic acid (GLA, 18:3 *n*-6), and EDA and negative for MA (*p* < 0.05). Interestingly, the amount of pups’ plasmatic GLA displayed the inverse behavior compared to EDA, by correlating positively with most milk SFA and negatively with milk MFA and PUFA. In addition, the amount of AA correlated negatively with the total *n*-3 FA (*p* < 0.05). 

**Figure 2:**
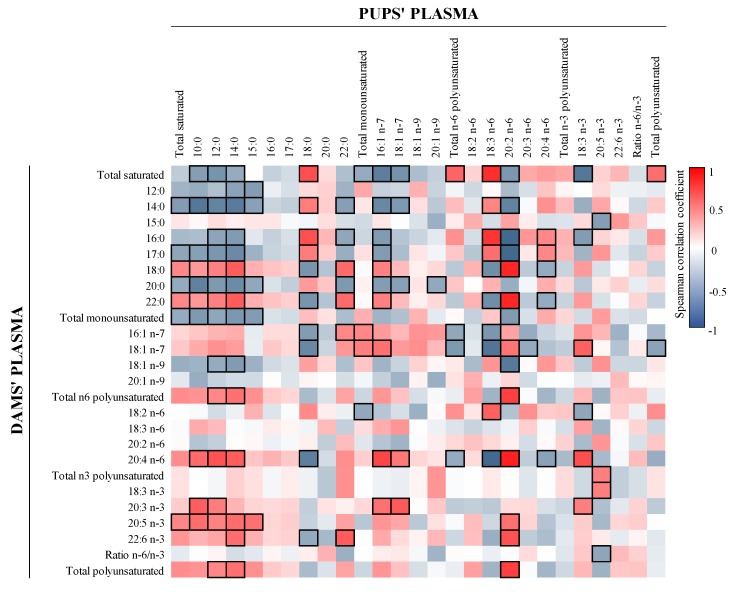
Correlations between dams’ and pups’ FA plasma content. The Spearman correlation coefficient is represented in the heat map following the color in the legends. Correlations with statistical significance (*p* < 0.05) are shown in a bold frame. Results derived from *n* = 6 for milk and dams’ plasma and *n* = 24 for pups’ plasma.

Finally, correlations between the plasma of the dams and pups were also studied (Figure 2), and, for example, plasma total SFA displayed negative associations with total MFA in pups and dams, respectively, and vice versa. In addition, EDA in pups’ plasma is positively associated with AA, total *n*-6 PUFA, and total PUFA from dams’ plasma.

### 3.2. Ig Profile

The concentration of the different Ig isotypes was determined in lactic serum and in the plasma of dams and pups (Table 2). The proportion of IgM, the first class of antibody to be produced during the primary immune response, decreased in the transmission line dam-milk-pup (~5%, ~1.5%, and ~0.5%, respectively; *p* < 0.05). IgG, which is involved in the secondary immune response, was the main Ig in all three samples, accounting for more than ~88% of the total in milk, ~93% in plasma of the dams, and ~98% in the plasma of the pups. 

The subtypes of IgG, IgG2b, and IgG2c, linked to a Th1 response, were the most abundant accounting for approximately 75% of all the Ig in the three types of samples. The highest proportion of IgG1 and IgG2a, subtypes linked to a Th2 response, was detected in the plasma of pups (~3% and ~17%, respectively). As a result, the ratio of Th1 and Th2 Ig was lower in the pups’ plasma compared to the dams’ and milk (~4, ~5, and ~6, respectively; *p* < 0.05). Finally, IgA, which is the Ig produced mainly in mucosal sites, differed substantially (*p* < 0.05) in proportion comparing the plasma of dams and pups (~2%) and milk samples (~10%).

The concentration of cytokines was assessed only in breast milk. The levels of IFNγ, TNFα, and IL-6 were under the limit of detection. The rest displayed low concentrations (0.19 ± 0.05 pg/mL of IL-4, 26.18 ± 7.22 pg/mL of IL-10, and 9.30 ± 6.35 pg/mL of IL-12).

**Figure 3:**
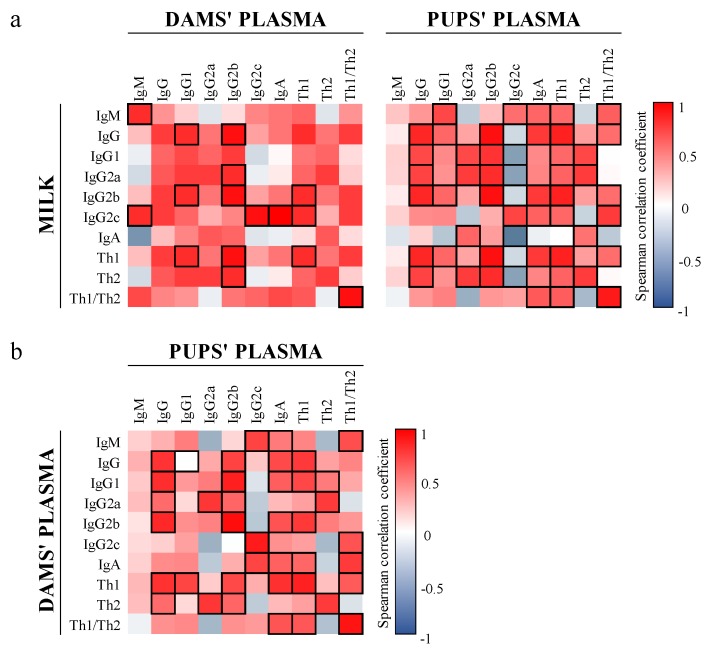
Correlation between the Ig composition of milk, dams’ plasma, and pups’ plasma. (**a**) Correlation between milk and dams’ and pups’ plasma and (**b**) correlation between dams’ and pups’ plasma. The Spearman correlation coefficient is represented in the heat map following the color in the legend. Correlations with statistical significance (*p* < 0.05) are shown in a bold frame. Results derived from *n* = 6 for milk and dams’ plasma and *n* = 16 for pups’ plasma.

Spearman correlation coefficient analysis revealed that the levels of Ig in plasma of dams and pups and in milk were strongly linked (Figure 3). There was a general dominance of positive correlations in all Ig. Particularly, we found a positive correlation between matching Ig in milk and dams’ plasma for IgM and Th1 Ig (IgG2b and IgG2c) and between matching Ig in milk and pups’ plasma for all Ig assayed (*p* < 0.05), in exception of IgM, IgG1, and IgA (Figure 3a). In addition, a strong association between matching IgGs and IgA was seen when comparing the plasma of the dams and the pups (*p* < 0.05, Figure 3b). Overall, the ratio Th1/Th2 showed a strong positive link in all comparisons (*p* < 0.05).

### 3.3. Microbiota Composition

**Figure 4:**
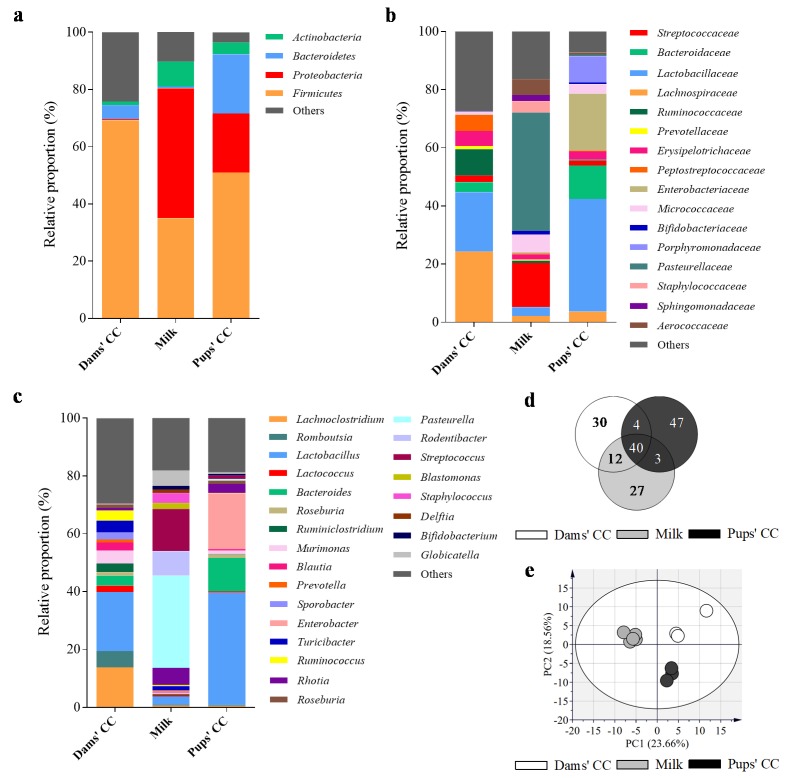
Assessment of microbiota composition of milk (*n* = 6) and CC (*n* = 3). The sequencing of the amplicon targeting the V3–V4 region of the 16S rRNA was performed following the 16S Metagenomic Sequencing Library Illumina 15044223 B protocol. The main taxonomic ranks abundances, corresponding to (**a**) phylum, (**b**) family, and (**c**) genera are represented in stacked bars. The qualitative assessment of microbiota was represented (**d**) in a Venn Diagram at the level of genera. Finally, the natural clustering of samples was assessed (**e**) by Principal Component Analysis (PCA) at the level of genera. Results derived from *n* = 5 for milk and *n* = 3 for both dams’ and pups’ CC. Cecal content (CC).

The microbiota composition was analyzed in CC of dams and their respective pups as well as in milk (Figure 4). As expected, the proportion of bacteria in the phylum level (Figure 4a) was different among the three types of samples. The CC of dams displayed the highest proportion of *Firmicutes* (69.2 ± 3.8%) followed by smaller proportions of *Bacteroidetes*, *Actinobacteria,* and *Proteobacteria* (4.9 ± 1.6, 1.3 ± 0.4 and 0.4 ± 0.1%, respectively). However, most of the bacteria in the milk samples belonged to the *Proteobacteria* phylum (45.4 ± 4.0%) and a lower proportion of *Firmicutes* and *Bacteroidetes* and higher of *Actinobacteria* were detected (35.0 ± 2.2, 0.7 ± 0.2, and 8.7 ± 2.6%, respectively). The CC of pups was more similar to the breast milk composition, characterized by a prominent *Proteobacteria* group (20.6 ± 2.7%), but containing higher proportions of *Firmicutes* (51.0 ± 5.5%) and specially *Bacteroidetes* (20.7 ± 5.5%).

Therefore, the microbiota composition at the level of family and genus was variable among the three types of samples analyzed (Figure 4b,c). The most abundant bacteria in dams CC samples belonged to the families *Lachnospiraceae*, *Lactobacillaceae,* and *Ruminococcaceae*, mainly represented by the genera *Lachnoclostridium, Lactobacillus,* and *Ruminococcus*, respectively. However, the milk samples were vastly dominated by *Pasteurellaceae* and *Streptococcaceae*, being the genera *Pasteurella* and *Streptococcus* the most abundant. The CC of pups displayed a higher content of *Lactobacillaceae* (i.e., *Lactobacillus*) compared to the CC of dams, and other families such as *Enterobacteriaceae* (i.e., *Enterobacter*) and *Bacteroidaceae* (i.e., *Bacteroides*) were highly represented. The content of *Bifidobacterium* was lower than 0.1% in the CC of dams and reached approximately 1.2 and 0.6% in the milk and pups, respectively.

The qualitative assessment of microbiota composition among the genera present in the samples was represented in a Venn Diagram (Figure 4d). A core of 40 genera was shared in all three compartments analyzed, accounting approximately for 70–90% of the microbiota composition. Specific genera were only detected in one of the compartments (30 in the CC of dams, 27 in milk, and 47 in the CC of pups). Interestingly, the milk displayed the highest proportion of exclusive genera, reaching approximately 4.3% of the microbiota. Three genera were only shared between milk and the CC of pups (*Corynebacterium*, *Haemophilus,* and *Mesocricetibacter*), four only overlapped in the CC of pups and dams (*Anaerobacterium*, *Paraeggerthella*, *Parasutterella,* and *Sporobacter*), and twelve others were exclusively shared between the CC of dams and milk (e.g., *Adlercreutzia*, *Erysipelaclostridium*, *Marvinbryantia,* and *Tyzzerella*).

Finally, the Principal Components Analysis (PCA) of the samples (Figure 4e) confirmed that the samples clustered apart depending on the type of compartment they belonged to.

### 3.4. Correlation between FA and Ig in Milk

**Figure 5:**
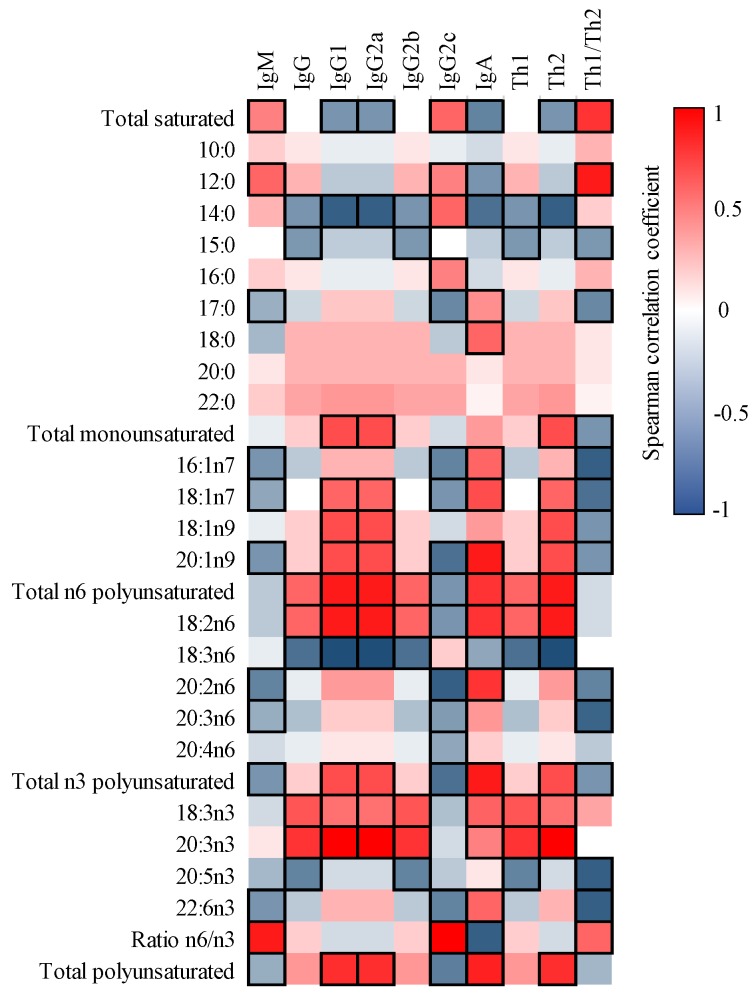
Correlation between the FA composition and Ig present in milk. The Spearman correlation coefficient is represented in the heat map following the color in the legend. Correlations with statistical significance (*p* < 0.05) are shown in a bold frame. Results derived from *n* = 6 for both milk and dams’ plasma.

In order to ascertain whether there was any relationship between the immune factors and the FA profile, the Spearman’s correlation coefficient was calculated among these variables and represented in a heat map (Figure 5). The amount of IgM inversely correlated with most of MUFA and PUFA, and displayed a positive correlation with the ratio *n*-6/*n*-3 (*r* = 0.9, *p* < 0.05). The IgG1, IgG2a, and IgA displayed a parallel pattern; they negatively correlated with total SFA (*r* = −0.60, *p* < 0.05), and they correlated positively with most MUFA and PUFA. In contrast, IgG2c displayed an opposite behavior. In connection with this, higher *n*-6 FA was associated with higher Th1 Ig, and higher total PUFA (both *n*-3 and *n*-6) and lower SFA with higher Th2 Ig.

### 3.5. Correlation between FA and Microbiota in Milk

**Figure 6:**
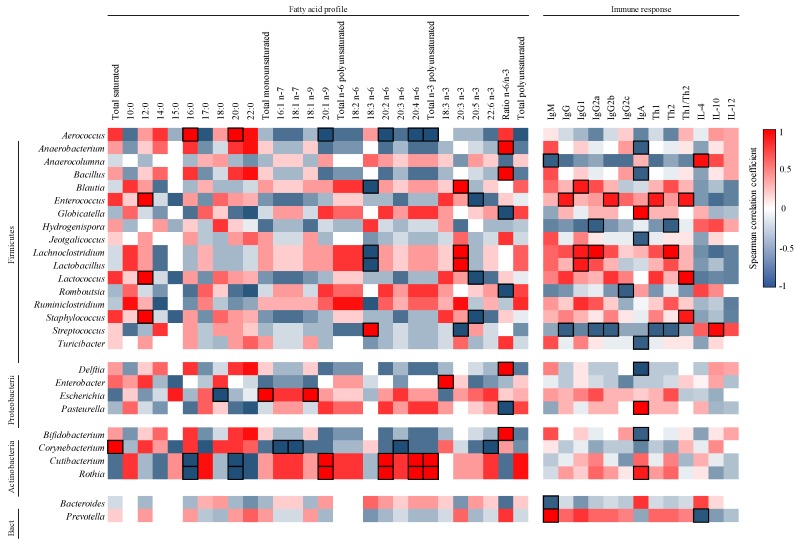
Correlation between the milk microbiota composition and the content of FA and Ig present in milk. The Spearman correlation coefficient is represented in the heat map following the color in the legend. Correlations with statistical significance (*p* < 0.05) are shown in a bold frame. Results derived from *n* = 5.

The milk composition was further analyzed by assessing the correlations between its content of FA and Ig with that of its microbiota (Figure 6).

In general, each genus showed a specific pattern of correlation and few significative associations appeared. For example, in relation to PUFA, the Firmicutes members *Lachnoclostridium* and *Lactobacillus* correlated negatively with GLA (*r* = −1.00, *p* < 0.05) and positively with eicosatrienoic acid (ETE, 20:3 n-3; *r* = 1.00, *p* < 0.05). In addition, other members of the Actinobacteria phylum, such as *Cutibacterium* and *Rhotia*, displayed negative correlations with the SFA of 16 and 20 carbons (*r* = −1.00, *p* < 0.05), and positive correlations with PUFA, such as EDA and AA (*r* = 1.00, *p* < 0.05). Overall, the ratio *n*-6/*n*-3 FA was strongly associated with many genera (*Anaerobacterium*, *Bacillus*, *Bifidobacterium, Delftia, Globicatella*, *Pasteurella,* and *Turicibacter*). 

### 3.6. Correlation between Ig and Microbiota in Milk

The correlation between Ig and microbiota in milk was analyzed and represented in a heat map (Figure 6). IgM was associated negatively with the proportion of *Anaerocolumna* and *Bacteroides* (*r* = −0.9, *p* < 0.05) and positively with *Prevotella* (*r* = 1.00, *p* < 0.05). On the one hand, Th1-type Ig positively correlated with the proportion of *Enterococcus* (*r* = 0.9, *p* < 0.05) and negatively with that of *Streptococcus* (*r* = −0.9, *p* < 0.05). On the other hand, Th2-type Ig correlated positively with *Lachnoclostridium* (*r* = 1.00, *p* < 0.05) and negatively with *Hydrogenispora* and *Streptococcus* (*r* = −0.9, *p* < 0.05). In regard to IgA, three genera (*Globicatella*, *Pasteurella* and *Rhotia*) displayed positive correlations and 6 other genera negative associations (*Anaerobacterium*, *Bacillus*, *Bifidobacterium*, *Delftia*, *Jeotgalicoccus,* and *Turicibacter*). Finally, in regard to the cytokines analyzed, positive correlations between *Anaerocolumna* and IL-4 (*r* = 0.95, *p* < 0.05) and between *Streptococcus* and IL-10 were found (*r* = 0.98, *p* < 0.05).

## 4. Discussion

There is little knowledge of the effect that maternal diet has on breast milk composition and the outcome in the offspring. As the rat is a common laboratory animal to perform nutritional intervention studies, the present study aimed to gain insight into the rat milk profile of FA, Ig, and microbiota and the association of these factors between the dam–pup pair. This knowledge may be used for further interventions having as objective the modulation of breast milk composition or just in the understanding of the relationship among them in this fluid. The results herein shed light on the degree of similarity between human and rat milk, supporting the use of rodent models for immunonutrition studies.

The overall FA composition of rat breast milk is similar to human breast milk, yet some differences were observed. Although some variability in FA proportions is found in humans depending on the geographic zone, overall rat breast milk seems to be richer in SFA, especially PA (~10% more), and weaker in MUFA, due to a lower proportion of OA (~10% less). The total proportion of PUFA is comparable (~20%), although the proportion of essential FA, such as LA and ALA, are known to be directly linked to the diet of the individual [27]. LC-PUFA, such as AA and DHA, are relevant for the development of brain and retina during pregnancy and lactation periods [28]. In this regard, the proportion of AA seems to be two times higher in rat breast milk; that of DHA is highly variable in humans, although the proportion of DHA (~0.2%) we found in rats is inside the range levels reported in human breast milk. Other LC-PUFA, such as EPA and *n*-3 docosapentaenoic acid (*n*-3 DPA, 22:5 *n*-3), display a similar proportion in both species [28,29,30,31,32,33].

The composition of human breast milk FA is strongly influenced by the diet of the mother, body stores, and genetics [34]. Moreover, in humans, breast milk is the major factor influencing the concentration of infant’s FA in plasma after birth [35]. For this reason, we also analyzed the FA profiles of dams’ and pups’ plasma in our preclinical approach. The overall plasma FA profile of dams and pups was very similar, suggesting that the composition of the milk generated in the dam was specifically tailored to transmit and reproduce a similar FA profile on their offspring. Nevertheless, the breast milk contained ~20% more SFA, evidenced by higher proportions of FA of 12, 14, and 16 carbons, and ~25% less PUFA, mainly linked to ~18% less AA and ~2% less *n*-3 LC-PUFA compared to plasma. Interestingly, although the amount of DHA in breast milk was approximately 9 times lower than in the plasma of dams, the amount reached in the pups’ plasma was 20 times higher compared to the breast milk and 2.5 times higher compared to the dams’ plasma. These facts evidence the bioconcentration of certain FA, such as AA and DHA, which requirements are of high importance for the newborn because their endogenous biosynthesis may be too immature to reach a normal development [7]. 

We also aimed to correlate the proportions of FA found in the different compartments in order to see if there were associations in the dam-milk-pup line, which might evidence an active transmission. Comparing dams’ plasma and milk FA, we did not observe significative associations, however, within all the FA analyzed, 11 of them displayed positive Spearman correlation coefficients (*r* > 0.3), whereas only 5 displayed negative coefficients (*r* < −0.3) and 4 showed no correlation (*r* ~0). When comparing milk with pups’ plasma, although milder correlations were observed due to high variability among litters, 7 FA displayed positive coefficients, 3 were negative, and 11 showed no correlation. Altogether, this data supports a link between the FA composition of milk and that of dams’ and pups’ plasma. However, other factors, such as internal body storages, FA metabolism, or high variability among litters, might be contributing to blur its direct association with the diet [4,5]. 

The immune factors present in milk, such as Ig, are important to confer passive immunity to the offspring. We analyzed the concentration of Ig in the three compartments and further searched for correlations. On the one hand, plasma of dams and pups displayed very similar amounts of IgG subtypes, and in similar proportion compared to milk, most likely because IgG molecules in suckling rats, but not in humans, are extensively absorbed in the intestine during lactation period [36,37]. In addition, this was also supported by the fact that every IgG subtype displayed significant positive correlations with their equal in all three compartments. Rat IgG subtypes can be classified depending on their skew toward Th1 (IgG2b and IgG2c) or Th2 (IgG1 and IgG2a). Likewise, although human subtypes are not homologous to those in rat, they have been grouped depending on whether their production arises from Th1 (IgG1, IgG3, and probably IgG2) or Th2 (IgG4) responses [38,39]. Accordingly, whereas Th2-type Ig are minority, Th1-type are dominant in breast milk of both rats and humans (>85% of the total IgGs), indicating a similar immunological function of breast milk in both species [40,41,42]. On the other hand, the IgM concentration in rat breast milk was similar to that reported in human milk [43]. Pups’ plasma displayed lower amounts of IgM as compared to dams’ plasma. IgM is the default Ig to be produced in primary responses and its endogenous production dominates in early life. At 14 days of life, IgM is low in the plasma of the pup because, contrary to IgG, it is not absorbed in the intestine and because their own immune system is still immature [20,37]. Indeed, no correlations were detected for IgM and their equals in the milk–pup or dam–pup pairs. 

Human milk is rich in IgA (~80%), the most abundant Ig in mucosal sites [44] reaching values of 300–2000 mg/L [45,46]. However, in rat breast milk, its amount (~57 mg/L) and proportion (~10% of the total) was lower than in humans because of the higher content of IgGs [37]. Whereas the biological role of IgGs in breast milk remains poorly studied, the transfer of IgA is biologically important, because it replaces the insignificant endogenous production of this Ig in the pup [47]. Milk IgA reaches the lumen and remains trapped in the mucous layer, playing a critical role in the exclusion of enteric pathogens as well as inducing immune tolerance and shaping the commensal microbiota [44,48]. No correlations between IgA in dam–milk or milk–pup were found, probably because IgA in the dam is produced directly in dimers from the secretory cells of the breast [49] and it is not absorbed by the suckling rat [50]. However, a significant correlation was seen between dams’ and pups’ plasma. The mechanism of this association remains unclear but may be linked to genetics or physiological factors related to immune homeostasis.

Breast milk also supports the initial intestinal colonization in early life [51]. In the present study, the rat milk microbiota was vastly dominated by *Proteobacteria* and *Firmicutes*, similarly to human breast milk [33]. It is difficult to perform a detailed comparison because human milk microbiota profiles are highly variable between women; they have been suggested to depend for instance on the feeding method, infant sex, obesity, and all other factors influencing the mother’s gut microbiota [52].

However, some differences in the genera present may be observed; whereas both displayed *Streptococcus* and *Staphylococcus*, rat milk showed considerable proportions of *Pasteurella, Rodentibacter, Rhotia,* and *Lactobacillus* and human milk *Serratia*, *Pseudomonas, Corynebacterium,* and *Ralstonia* [53]. 

From the 86 genera present in dams’ CC and 82 in the milk, 52 were shared. This high overlapping could be linked with the mechanism of uptake and transmission of intestinal bacteria toward breast milk, known as entero-mammary route, which has been hypothesized in recent years [54]. Moreover, the CC of pups shared 43 genera out of 94 with the breast milk, from which only three genera were not found in the dams’ CC (*Corynebacterium*, *Haemophilus,* and *Mesocricetibacter*). These results suggest that breast milk could be actively involved in the transmission of the intestinal microbiota of the dam to the pup.

The gut microbiota composition of 14-day old rats was similar to in the study of Marungruang et al. (2018), where the most abundant phyla were *Proteobacteria*, *Firmicutes,* and *Bacteroidetes* [55]. Interestingly, high abundances of *Proteobacteria* were seen in milk and pups’ CC, but not in the intestinal microbiota of dams. In fact, *Proteobacteria* are abundant in early life because they consume oxygen and make the habitat suitable for strict anaerobes; as a consequence, their proportion decreases throughout life [55,56]. Although some differences are found between rats and humans, the study of Flemer et al. (2017) concluded that the intestinal microbiota of rats was closer to humans than that of mice, supporting the use of this model in this context [57] and, therefore, the need of the results presented here.

FA are known to have direct effects on the immune system. Therefore, the high content of lipids in breast milk may exert multiple immunomodulatory effects on the newborn [14]. While preclinical studies have shown the limited impact of SFA on the immune system, PUFA have the ability to modulate lymphocyte functions and antibody responses [10]. In fact, in the present study, most of the correlations between milk FA and Ig were found in PUFA, suggesting the key link between PUFA and the immune system. 

The total *n*-3 PUFA in milk negatively correlated with IgG2c and positively correlated with IgG1 and IgG2a, supporting their anti-inflammatory capacity to block Th1 and enhance Th2 responses [58,59]. Precisely, EPA and DHA seemed to be linked to lower amounts of Th1 Ig, thus negatively correlating with the Th1/Th2 ratio. On the contrary, ALA and ETE were associated with higher amounts of Th2 Ig. Accordingly, other studies found that EPA and DHA can reduce de production of IL-2 in splenocytes [60] or IFNγ and IL-1 in gestating women [61], which might be blocking the production of Th1 Ig. Overall, the ratio of Th1/Th2 Ig in milk correlated positively with the *n*-6/*n*-3 ratio indicating, as expected, that the higher the levels of the more pro-inflammatory PUFA (i.e., *n*-6), the higher the levels of the Ig isotype derived from a pro-inflammatory activation (i.e., the Th1 response). In line with our results, it has been described that dietary *n*-6/*n*-3 ratio influences the production of Ig in colostrum of lactating sows impacting their levels in plasma of suckling piglets [62].

Finally, the correlations between the milk microbiota and its content in FA and Ig were analyzed. Dietary FA can impact the intestinal microbiota composition [63], and vice versa, the metabolites produced by the microbiota can have an impact on lipid metabolism [64]. Recent studies found associations between the FA profile and the microbiota composition in human milk [33,52]. In our context, although we did not detect a lot of significative correlations, we identified that the ratio *n*-6/*n*-3 FA seemed to be influencing the proportion of several genera belonging to diverse phyla. In this regard, most genera displayed a positive correlation, evidencing that higher *n*-6 and lower *n*-3 FA promoted higher abundances of, for example, *Bifidobacterium*, *Aerococcus,* or *Delftia.* Interestingly, Kumar et al. (2016) also found a negative association with *n*-3 FA in milk and *Bifidobacterium* [33]. In addition, we found a negative correlation between the amount of GLA and the proportion of *Lactobacillus* in milk. This result is in accord with the fact that certain PUFA, such as GLA, can inhibit the growth of some *Lactobacillus* species when added in the growth media [65].

On the other hand, the crosstalk between the intestinal microbiota and the immune system is pivotal for the maturation of the immune system and the acquisition of oral tolerance in early life because the microbiota gives specific signals to train the immune system [66]. Moreover, several factors related to the immune system, such as the production of sIgA, has a huge impact on the colonization of bacteria and the shaping of the intestinal microbiota [66]. Herein, the proportion of breast milk *Enterococcus* correlated positively with Th1 Ig (the IgG2b subtype). This result is in accordance with the fact that, for example, the administration of *Enterococcus faecalis* to mice under different experimental conditions is able to influence the pattern of cytokines toward the Th1 phenotype [67,68,69]. In addition, we observed that the IgA correlated positively with the most abundant genera in milk, such as *Pasteurella* or *Globicatella*. In contrast, negative correlations were found with minor populations, such as *Bifidobacterium* or *Turicibacter*. To our knowledge, this is the first time to describe such type of associations that need to be further explored.

It is also accepted that maternal exposure to bacteria may influence cytokine composition of breast milk [70]. In our study, *Streptococcus* in breast milk correlated positively with the amount of IL-10 in this fluid. This result is in accord with a study of Doare et al. (2017), who found that colostrum received by infants colonized with Group B *Streptococcus* (GBS) had higher levels of IL-10 than those who tested negative for GBS [70]. 

## 5. Conclusions

In summary, we established the profile of FA, Ig, and the microbiota composition of rat breast milk. Moreover, we found many associations of these factors in the dam–pup pair. The results herein might indicate an active transfer of FA, Ig, and microbiota through breast milk, although further research should be performed to confirm that. The recommendation of dietary interventions during pregnancy and/or lactation that have an impact on one of these factors may also influence the others and, therefore, the overall immune quality of breast milk. Hence, it would be a useful strategy to promote immune development and provide higher passive protection against pathogens to the newborn. Overall, this study supports the use of the rat model as a successful approach to study these strategies at a preclinical level as an initial translational approach.

## Figures and Tables

**Table 1:** Fatty acids (FA) composition of milk and plasma of dams and pups.

(%)	Dams’ Plasma	Milk	Pups’ Plasma
**Total saturated**	40.93 ± 0.27	63.88 ± 1.69	42.50 ± 0.44
8:0	-	2.06 ± 0.17	-
10:0	-	7.90 ± 0.42	0.72 ± 0.07
12:0	0.47 ± 0.03	9.53 ± 0.37	2.19 ± 0.13
14:0	0.59 ± 0.03	12.47 ± 0.57	3.95 ± 0.16
15:0	0.24 ± 0.01	0.14 ± 0.01	0.17 ± 0.01
16:0	21.18 ± 0.47	28.22 ± 0.89	24.62 ± 0.29
17:0	0.38 ± 0.01	0.15 ± 0.01	0.24 ± 0.01
18:0	17.27 ± 0.24	3.23 ± 0.06	10.12 ± 0.19
20:0	0.32 ± 0.01	0.12 ± 0.01	0.22 ± 0.01
22:0	0.47 ± 0.06	0.06 ± 0.01	0.27 ± 0.01
**Total monounsaturated**	15.35 ± 0.77	16.31 ± 1.21	10.73 ± 0.38
16:1 *n*-7	1.37 ± 0.12	1.11 ± 0.23	0.51 ± 0.02
18:1 *n*-7	1.24 ± 0.09	1.02 ± 0.16	0.96 ± 0.02
18:1 *n*-9	12.49 ± 0.70	13.69 ± 0.85	8.90 ± 0.37
20:1 *n*-9	0.28 ± 0.02	0.23 ± 0.03	0.36 ± 0.10
**Total *n*-6 polyunsaturated**	43.73 ± 0.84	18.39 ± 0.55	41.42 ± 0.41
18:2 *n*-6	21.10 ± 0.76	16.09 ± 0.40	20.67 ± 021
18:3 *n*-6	0.64 ± 0.06	0.34 ± 0.02	0.49 ± 0.02
20:2 *n*-6	0.30 ± 0.07	0.39 ± 0.07	0.30 ± 0.02
20:3 *n*-6	-	0.33 ± 0.03	1.09 ± 0.04
20:4 *n*-6	18.34 ± 0.89	0.93 ± 0.05	19.97 ± 0.43
22:4 *n*-6	-	0.24 ± 0.05	-
22:5 *n*-6	-	0.07 ± 0.01	-
**Total *n*-3 polyunsaturated**	3.35 ± 0.20	1.30 ± 0.05	3.26 ± 0.11
18:3 *n*-3	0.38 ± 0.06	0.90 ± 0.02	0.32 ± 0.02
20:3 *n*-3	1.34 ± 0.20	0.05 ± 0.01	-
20:5 *n*-3	0.52 ± 0.03	0.11 ± 0.01	0.16 ± 0.02
22:5 *n*-3	-	0.10 ± 0.02	-
22:6 *n*-3	1.11 ± 0.10	0.13 ± 0.01	2.78 ± 0.12
**Ratio *n*-6/*n*-3**	12.18 ± 0.58	14.14 ± 0.12	12.96 ± 0.37
**Total polyunsaturated**	43.73 ± 0.84	19.69 ± 0.59	45.77 ± 0.47

Results are expressed as mean percentage of total FA ± S.E.M. Results derived from *n* = 6 for dams and *n* = 24 for pups.

**Table 2:** Analysis of absolute and relative Ig concentration in milk and plasma of dams and pups.

	Dams’ plasma		Milk		Pups’ Plasma	
	µg/mL	%	µg/mL	%	µg/mL	%
**IgM**	191.7 ± 36.5	4.8 ± 1.0	9.5 ± 2.0	1.6 ± 0.2	18.8 ± 0.6	0.5 ± 0.0
**IgG**	4139.7 ± 969.6	92.94 ± 1.1	589.1 ± 120.4	88.5 ± 1.6	4207.3 ± 400.7	97.9 ± 0.1
**IgG1**	93.7 ± 9.7	2.37 ± 0.3	9.6 ± 1.0	1.6 ± 0.2	125.3 ± 6.2	3.1 ± 0.2
**IgG2a**	576.5 ± 66.3	14.89 ± 2.3	70.1 ± 7.3	12.0 ± 1.8	682.3 ± 35.5	17.2 ± 1.3
**IgG2b**	2425.3 ± 779.5	50.56 ± 5.6	362.6 ± 102.5	50.0 ± 6.7	2534.4 ± 372.0	55.5 ± 3.4
**IgG2c**	1044.1 ± 199.8	25.12 ± 4.4	146.8 ± 24.0	24.9 ± 5.3	865.3 ± 88.3	22.1 ± 3.0
**Th1/Th2 ^a^**	5.1 ± 1.0	5.06 ± 1.0	6.3 ± 1.2	6.3 ± 1.2	4.2 ± 0.4	4.2 ± 0.4
**IgA**	90.1 ± 9.8	2.26 ± 0.2	57.4 ± 6.6	10.0 ± 1.6	65.7 ± 2.9	1.6 ± 0.1

Results are expressed as mean ± S.E.M and relative percentage. ^a^ Th1/Th2 ratio refers to the relationship between IgG2b + IgG2c and IgG1 + IgG2a, respectively. Results derived from *n* = 6 for dams and *n* = 16 for pups.
